# Ability and willingness to pay for family planning services in low resource settings: evidence from an operational research

**DOI:** 10.4314/ahs.v22i1.5

**Published:** 2022-03

**Authors:** Nazarius Mbona Tumwesigye, Fredrick Makumbi, Aggrey Mukose, Lynn Atuyambe, Cissie Namanda, Sarah Ssali, Ritah Tweheyo, Andrew Gidudu, Carole Sekimpi, Catherine Verde Hashim, Martha Nicholson, Ritah Nakigudde Waddimba, Peter Ddungu

**Affiliations:** 1 Makerere University School of Public Health, Department of Epidemiology and Biostatistics; 2 Makerere University School of Public health, Department of Community health and behavioural sciences; 3 Makerere University, School of Women and Gender Studies; 4 Marie Stopes International; 5 FCDO, Uganda

**Keywords:** Ability-to-pay, willingness-to-pay, total market approach, market segmentation, family planning

## Abstract

**Objective:**

This paper establishes levels and patterns of ability and willingness to pay (AWTP) for contraceptives, and associated factors.

**Study design:**

A three-stage cluster and stratified sampling was applied in selection of enumeration areas, households and individuals in a baseline survey for a 5-year Family planning programme. Multivariable linear and modified Poisson regressions are used to establish factors associated with AWTP.

**Results:**

Ability to pay was higher among men (84%) than women (52%). A high proportion of women (96%) and men (82%) were able to pay at least Ug Shs 1000 ($0.27) for FP services while 93% of women and 83% of men who had never used FP services will in future be able to pay for FP services costed at least Shs 2000 ($0.55). The factors independently associated with AWTP were lower age group (<25 years), residence in urban areas, attainment of higher education level, and higher wealth quintiles.

**Conclusion:**

AWTP for FP services varied by different measures. Setting the cost of FP services at Shs 1000 ($0.27) will attract almost all women (96%) and most of men (82%). Key determinants of low AWTP include residence in poor regions, being from rural areas and lack of/low education.

Implications statement: Private providers should institute price discrimination for FP services by region, gender and socioeconomic levels. More economic empowerment for disadvantaged populations is needed if the country is to realise higher contraceptive uptake. More support for total market approach for FP services needed.

## Introduction

Ability and willingness to pay (AWTP) are two different concepts that are often assumed to be synonymous^1^. Ability to pay (ATP) can be defined as the capability to pay for a service with respect to a person's income^2^ while Willingness to pay (WTP) can be defined as the maximum amount an individual is willing to pay for a good or a service 3. Assessment of AWTP helps to characterise users and estimate effective demand. Such evidence can inform the design of innovative interventions appropriate for specific contexts and population segments to enhance use of FP services and better fertility outcomes 4,5.

Little is known about the level and correlates of AWTP for FP services. Early studies showed that 77% of Ugandans could not afford to pay for FP commodities^6^. Elsewhere, factors associated with AWTP for FP services include poverty and inequality^7^, price of FP commodities 8, availability of free FP services8, 9, female gender10 and lack of sensitization on payment for FP services11. Education and occupation have been significantly linked to willingness to pay for health services in general^12^, ^13^.

Uganda is in its initial stages of development of a strategic plan for Total Market Approach (TMA) to provision of family planning services. TMA, commonly referred to as whole market, is system in which all sectors-public, commercial and non-governmental organisation or donor funded social marketing are integrated within one market segmented by ability and willingness to pay^14^. The ultimate goal of TMA is to create an efficiently segmented market that provides access to a full range of family planning products and services^15^. TMA approach is best for countries with uneven economic growth and most of the people heavily reliant on subsidised or free services^16^. Uganda is one of such countries with a strong economic growth^17^ but with rising inequality depicted by high Gini-coefficient of 0.43 18, 19. New evidence on levels, differential and correlates of AWTP will feed into the TMA implementation.

## Methods

### Design

A cross-sectional survey was conducted in 7 of the 10 statistical regions of Uganda as per 2011 Uganda Demographic and Health Survey (UDHS) 20. This was at the start of a program entitled “Reducing High Fertility Rates and Improving Sexual Reproductive Health Outcomes in Uganda (RISE)”. The regions were purposively selected and they were: Western, Central 1, Central 2, East Central, Eastern, Karamoja and West Nile. The data collection period was August-September 2019 and it targeted women and men in age groups 15–49 and 15–54, respectively. A three-stage cluster and stratified sampling was applied in selection of the sample. The three stages were the enumeration area (EA), household, and a household member while the strata were the selected regions. Uganda Bureau of Statistics (UBOS) drew a random sample of EAs in each of the seven regions while research assistants sampled households and household members.

The sample size was estimated from the same formula used for the UDHS21 and Performance Monitoring and accountability 2020 (PMA2020)^22^. With a design effect of 2, desired margin of error () 0.04, the individual response rate at 80% and household response rate of 80%, and intention to use of 62% 23 a sample size of 1,767 was computed. For both men and women this was 3,534 (2x1,767) and 505 per region. This translated to 3607 after adjusting for a non-response rate of 2% reported in UDHS 2016. The number of people successfully interviewed were 1250 males and 1346 females. This made a total of 2596 which was 72% of the targeted 3607. The 28% non-response was largely due to the rainy season of August-September and the design that did not allow substitution of respondents nor switching interviewers when an eligible respondent was of a gender different from that of interviewer. Nevertheless, the implementation followed strict UBOS and PMA20^20^ guidelines and this resulted into a sample that had similar background characteristics with that of UDHS 2016 and had high precision for key some of the FP indicators.

### Data collection

Maps of the selected Enumeration Areas (EAs) were obtained from UBOS and used to locate the selected EA boundaries. Using the maps EA boundaries were identified and households therein listed. A sample of 60 households was drawn from each EA using a random number generator app with the first 30 households allocated to male and the next allocated to the female data collector. Each research assistant was assigned to interview a respondent of the same sex to improve quality of data An EA with less than 120 households was annexed to an adjacent EA whose main entrance was the closest. An EA main entrance referred to the first point where the main road first connects with the EA from whichever direction. Details of all the annexed and parent EAs were sent to UBOS for updating of EA sampling probabilities. The realised cluster size ranged from 26 to 58 households.

In each household, the details of name, index number, age and sex were entered into the pre-programmed listing form within the ODK online data collection software^24^. A random selection of one eligible participant per household was carried out using a code developed within the ODK's programming enabling option. The selection of households followed a non-substitution policy that did not allow replacement if respondents were unavailable^25^ even after 3 call backs.

### Measurements

Ability-to-pay (ATP) was measured by percent of respondents that used their own money to access contraception services or found it easy to get the money or would pay a higher price if the costs were increased. Another measure of ATP was the amount one paid for the current FP method and amount one would pay for FP service in the future if he/she never used the services. Willingness-to-pay (WTP) was measured by the percent of those that currently received free FP services but were willing to pay for the same services in future and percent of those who never used FP services but were willing to pay for the services in future.

### Data management and analysis

The ODK online data collection platform was fitted with range and consistency checks and residual cleanup was carried out using STATA V14 software. Individual sampling weights were computed as inverse of the product of probabilities for selection of EAs, households within each EA and an eligible participant within the EA, and response rates for households with an eligible participant.

To adjust for design effects of the survey the svyset command was applied specifying the sampling weight, stratum of regions and enumeration area (EA) Code. The weighted cross-tabulations of ability and willingness to pay indicators and background characteristics were computed to show the levels and patterns of ATP and WTP for FP services. Multivariable linear analysis for natural log of the maximum amount of money respondents were able to pay for FP services and modified Poisson regression (MPR) for willingness to pay were used for advanced analysis. MPR models for willingness to pay were preferred to logistic regression because the later technique tends to overestimate the effects of the association when the outcome prevalence is 10% or higher 26. Beside the background characteristics, the selection of the variables to include in the models was guided by key monitoring variables of the RISE program. For example, disability status was important because the program monitors access to family planning services by vulnerable populations.

For presentation of results reverse cumulative distribution of amount paid for last FP services is used to assess ATP. The reverse cumulative distribution graph completely displays all the data, allows a rapid visual assessment of important details of the data^27^. The horizontal axis represents the amount paid for last FP services and the vertical scale represents the percent of respondents that paid at least that amount. The plot created reverses the approach of the cumulative distribution graph which plots a value against the percentage of equal or less values28. It's for this reason the curve is known as the reverse cumulative distribution (RCD) curve^27^.

## Results

### Levels of ability to pay

Table 1 shows levels of ability to pay (ATP) for FP services among men and women by background characteristics. A higher proportion (84%) of men used their own money to pay for FP services compared to women (52%). The difference was evident across age groups, marital status, other background characteristics and type of current FP method used. Among men and women, use of own money to pay for FP services was lowest among rural residents and students.

**Table 1:** Ability to pay: Percent of FP users that paid their own money, able to get the money, and amount they paid for the services

Characteristics	Men					Women				
	n- (weighted)	Used own money %)	getting money was easy/very easy (%)	Would pay higher if price increase d by 10% (%)†	Amount you paid for last FP services median (IQR) in '000 of Ug Shs#	n- (weightet)	Used own money (%)	getting money was easy/very easy (%)	Would pay higher if price increased (%)†	Amount you paid for last FP services median (IQR) in '000 of Ug Shs#
**Age**										
15–19	39	78.5	56.4	58.6	1.0(0.5–5.0)	42	32.5	73.1	68.3	2.3 (2.0–5.0)
20–24	57	80.4	71.9	82.5	2.0(0.8–5.0)	105	39.7	58.9	78.1	3.0 (2.0–5.5)
25–29	63	91.4	74.0	80.0	2.0(1.0–4.0)	133	58.8	70.1	83.5	3.0 (2.0–5.0)
30–34	58	89.2	68.2	79.1	3.0(2.0–8.0)	80	54.1	67.5	69.6	3.0 (2.5–8.0)
35–39	48	81.7	75.8	88.5	5.0(1.0–10.0)	76	69.9	67.8	90.0	2.0 (2.0–4.0)
40–44	40	80.0	62.0	75.0	3.0(2.0–5.0)	27	68.6	71.3	71.6	3.0 (1.5–4.5)
45–49	33	84.2	66.1	73.2	5.0(2.0–10.0)	30	45.8	72.0	95.4	2.5 (1.5–5.0)
50–54	21	57.7	46.1	100.0	3.0(2.0–20.0)					
**Marital status**										
Single	75	85.3	71.6	71.4	1.5(0.5–4.0)	48	47.8	48.1	80.5	2.8 (2.0–6.0)
Married/cohabiting	269	82.9	65.9	81.1	3.0 (1.5–7.0)	398	47.2	70.4	79.4	5.0 (2.0–5.0)
Widow/separate/divorced	15	85.6	89.3	87.0	1.0(1.0–1.5)	46	81.1	68.1	77.0	3.0 (1.5–5.0)
**Residence**										
Urban	67	87.3	75.4	82.1	2.0 (0.5–5.0)	78	62.2	73.0	81.5	5.0 (2.0–10.0)
Rural	292	80.7	63.1	76.4	3.0 (1.0–5.0)	413	46.1	63.5	78.1	3.0 (2.0–5.0)
**Level of** **Education**										
None	13	77.3	56.1	100.0+	5.0 (3.0–6.5)	59	57.8	54.3	82.0	5.0 (2.0–10.0)
Primary	214	80.7	60.9	78.2	2.0 (1.0–5.0)	290	53.8	67.5	78.5	3.0 (2.0–5.0)
Secondary	109	85.5	74.1	79.0	2.0 (1.0–7.0)	126	50.0	71.7	81.3	3.0 (2.0–7.0)
Tertiary	23	94.7	91.0	74.0	4.5 (1.5–5.0)	18	41.2+	56.5	59.5+	20 (10.0–32.5)
**Region**										
Central1	58	82.8	79.6	85.3	3.5 (1.3–8.5)	57	60.9	69.8	75.7	5.0 (2.0–7.0)
Central2	45	84.2	65.7	93.9	3.5 (2.0–10.0)	59	43.7	58.7	77.0	3.0 (2.0–5.0)
Eastern	66	79.6	54.1	63.8	2.0 (1.0–5.0)	97	37.0	57.5	79.8	2.5 (2.0–5.0)
East Central	40	87.2	68.0	78.2	2.0 (1.0–4.0)	89	54.0	76.6	86.6	2.0 (1.3–2.3)
Karamoja	27	100.0+	68.2	61.7	5.0(2.0–10.0)	14	30.4	15.2	75.0	3.0 (2.0–6.0)
Western	91	76.9	50.3	59.0	3.0 (1.0–5.0)	138	51.4	69.5	82.0	3.0 (2.0–6.0)
West Nile	33	99.2	95.1	95.2	0.5 (0.5–0.5)	37	53.9	81.6	92.8	3.0 (1.0–25.0)
**Wealth Quintile**										
Lowest	117	86.7	58.3	68.4	2.0 (1.0–5.0)	126	47.3	61.5	71.2	3.0 (2.0–4.0)
Second	47	83.2	57.4	74.2	1.8 (0.5–5.0)	64	50.3	57.6	70.8	3.0 (1.5–4.0)
Middle	67	80.2	76.4	87.3	3.0 (1.0–7.0)	113	64.1	69.0	84.3	3.0 (2.0–5.0)
Fourth	113	83.0	66.8	77.2	3.0 (1.0–7.0)	146	43.8	67.3	81.8	3.0 (2.0–7.0)
Highest	16	100.0	100.0	94.3	2.0(1.0–7.0)	42	57.5	73.2	78.1	5.0 (2.5–10.0)
**Occupation**										
Unemployed	11	81.7	44.4	38.0	3.0(0.5–15)	83	56.7	68.6	64.4	3.0 (2.0–5.0)
Employed	323	84.5	65.6	80.9	3.0 (1.0–5.0)	399	51.5	69.2	84.0	3.0 (2.0–5.0)
Student	23	73.3	64.1	84.2	1.0 (0.5–5.0)	10	40.8	28.1	90.0	4.0 (2.0–15.0)
**Disability status**										
no difficulty in all domains	261	84.2	70.4	79.8	3.0 (1.0–7.0)	330	53.5	69.2	81.3	3.0(2.0–7.0)
A lot of difficulty /unable to function in at least one domain	98	82.0	63.1	75.9	2.0 (1.0–5.0)	162	49.7	63.1	75.5	3.0 (2.0–5.0)
**Number of living** **children**										
0	39	94.4	88.1	88.2	1.0(0.5–5.0)	12	17.0	43.4	64.7	2.0 (2.0–5.0)
1	39	76.4	73.2	93.2	2.0(1.0–6.0)	76	52.0	74.1	87.7	4.8 (2.8–6.5)
2	37	86.2	57.8	60.8	4(2.0–10.0)	90	58.4	72.8	77.8	3.0 (2.0–5.0)
3	51	96.2	73.1	86.4	3.0 (1.0–7.0)	75	46.9	74.6	76.0	3.0 (2.0–5.0)
4+	155	76.2	62.3	81.8	3.0(1.5–7.0)	220	58.6	65.7	79.9	3.0 (2.0–5.0)
**Method** **currently use**										
IUD	16	89.2	80.0	85.5	11(5.0–20.0)	12	34.5	63.2	86.0	17.5(7.0–30.0)
Implants	42	82.0	63.9	77.1	10.0(10–20)	76	43.7	65.2	73.0	7.5(4.5–10.0)
Injectables	35	80.8	58.5	72.6	5.0(3.0–10.0)	90	52.2	74.1	80.5	5.0(3.0–7.0)
Male condoms	74	92.8	78.0	76.1	1.0 (0.5–2.5)	75	11.0	49.9	78.3	2.0 (1.0–5.0)
Pills	14	68.4	61.5	67.0	3.0(2.0–5.0)	220	68.7	71.8	79.0	2.3 (1.5–10.0)
**Total**	359	83.6	68.5	78.9	3.0 (1.0–8.0)	492	52.2	67.1	79.4	3.0 (2.0–5.0)

† Applies to only those that had paid for FP services

# Considered exchange rate of $1.00=3650 Uganda shillings.

* The question is asked to only those th at did not pay for their last FP services.

+ few observations- only 8 men had paid for the last FP method taken, only 8 had no formal education and only 15 men answered the question on future use of FP services.

- no observations/not applicable

Over two thirds of the men (69%) and women (67%) found it easy to get the money they used to pay for the FP services. Among men, the proportion rose from 56% in age group 15–19 to 76% in age group 35–39 but declined to 46% in the last age group. Among women, the proportion remained high (>67%) for all age groups except for those in age group 20–24 (59%). Other categories with lower proportion that found it easy to get the money to pay for FP services were the married/cohabiting, rural residents, the uneducated, those in eastern and western regions, the poor in first and second wealth quintile, the unemployed, those with a disability, those with at least four children and those who currently use injectables. Another measure of ability to pay (ATP) was whether respondents would pay higher if the price was increased by 10%. The same pattern of ATP was observed by different socio-economic characteristics.

Overall, the median amount of money paid for last FP services used was Shs 3000 ($0.82) and it's the same for both men and women. Among men, the median payment was lowest among the youngest (Ug Shs 1000/$0.27), the single (Ug Shs 1500/$0.42), the students (Ug Shs 1000/$0.27), the disabled (Shs 2000/$0.56), those without children (Shs 1000/$0.27) and those who took male condoms at last FP service visit, residents of West Nile (Shs 500/$0.14), Eastern (2000/$0.55) and East Central Shs 2000/$0.56). For women, the median payment was lowest among those who were single (Ug Shs 2800/$0.46), rural (Ug 3000/$0.82), resident of east central (2000/$0.55) and those who used male condoms at last FP use (Ug Shs 2000/$0.55). A similacomputation for the amount of money respondents will pay in future for the same FP services showed a similar pattern.

**Figure 1:**
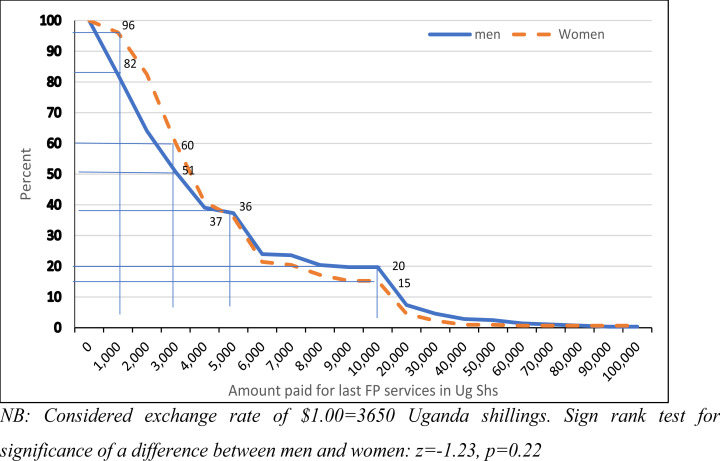
Reverse Cumulative distribution plot: Percent of respondents that paid a given amount of money or more for FP services they last received

Figure 1 shows the proportion of respondents that paid a given amount of money or more for the FP services they last received. A higher proportion of men paid more than Shs 5000($1.40) for FP services than women and it's the reverse for FP services that cost less than Shs 5000. The figure further shows 96% of women paid at least Shs 1000 ($0.27) for FP services but this number reduced to 15% for services costing at least Shs 10,000($2.80). A lower proportion of men (82%) paid for services costing at least Shs 1000 ($0.27) but a higher proportion (20%) paid for services costing at least Shs 10,000($2.74).

**Figure 2:**
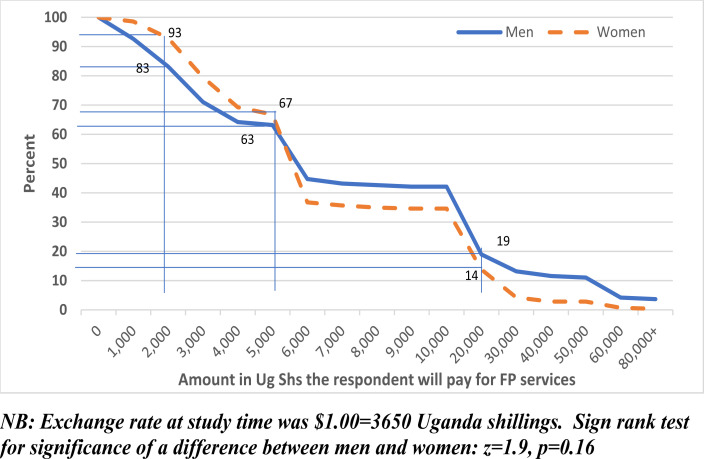
Reverse Cumulative distribution plot: Percent of respondents who had never used FP before that will pay a given amount of money or more for FP services in future

Respondents who had never used FP were asked the maximum amount of money they could afford to pay for FP services. Figure 2 shows the proportion that will afford to pay a given amount of money or more for FP services among respondents that had never used the services before. For example, while 93% of women and 83% of men will afford to pay at least Shs 2,000 (USD 0.55) for FP services only 19% of men and 14% of women will afford to pay at least Shs 20,000 (USD 5.48) for FP services. When the cost of FP services is less than Shs 5000 a higher proportion of women can afford to pay compared to men while it's the reverse with higher cost.

**Figure 3:**
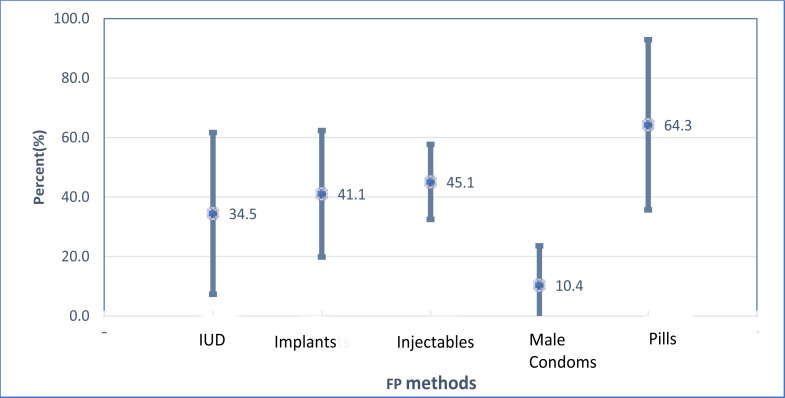
Ability to pay: Percent of Married/cohabiting women that used their own money to pay for different kinds of FP methods

Figure 3 shows the percent of married/cohabiting women that used their own money to pay for FP services by the FP method they were using at the time of the study. Use of own money to pay for FP services was most prevalent among pill users (64%) followed by injectable users (45%) although the prevalence is less precise in the former than in the latter.

### Levels of Willingness to pay

Overall, two thirds of the men (66%) and women (67%) were willing to pay for the same FP services in future (Table 2). For both men and women WTP for the same FP methods in the future was lowest among the single (men,61%; women, 63%), those without formal education (men, 58%; women, 64%), and residents of Karamoja region (Men, 25%; Women, 43%). WTP for the same services in future did not change much by type of residence and FP method currently used.

**Table 2:** Willingness to pay: Percent of FP users that were willing to pay for FP services in future

Characteristics	Men			Women		
	n- (weighted)	Willing to pay for the Method used In future (%) *	Never used FP but willing to pay for FP services in future (%)	n (weighted)	Willing to pay for the method used in future (%) *	Never used FP but willing to pay for FP services in future (%)
**Age**						
15–19	39	63.2	43.9	42	72.2	52.5
20–24	57	62.7	57.5	105	67.8	56.1
25–29	63	66.7	57.8	133	72.7	40.7
30–34	58	71.6	48.6	80	67.5	31.2
35–39	48	58.5	66.9	76	60.7	44.1
40–44	40	62.7	38.6	27	67.6	45.3
45–49	33	72.3	30.0	30	52.2	33.4
50–54	21	75.6	35.3	--		
**Marital status**						
Single	75	60.7	44.9	48	62.5	48.2
Married/cohabiting	269	67.1	51.4	398	68.3	47.2
Widow/separate/divorced	15	73.0	53.5	46	64.8	42.6
**Residence**						
Urban	67	65.1	55.9	78	48.8	47.7
Rural	292	66.2	47.3	413	70.9	46.9
**Level of Education**						
None	13	58.3	46.3	59	64.2	19.1
Primary	214	60.9	46.0	290	68.6	53.0
Secondary	109	77.6	54.5	126	68.8	56.2
Tertiary	23	62.6	75.2	18	47.9+	86.2
**Region**						
Central1	58	56.4	50.0	57	42.2	56.9
Central2	45	83.7	50.9	59	68.3	66.4
Eastern	66	61.7	37.9	97	75.7	48.9
East Central	40	64.6	45.4	89	72.8	41.1
Karamoja	27	25.4	45.0	14	43.0	20.9
Western	91	77.0	59.3	138	72.6	49.0
West Nile	33	71.6	50.4	37	60.3	46.4
**Wealth Quintile**						
Lowest	117	60.8	45.3	126	56.9	28.5
Second	47	69.8	43.7	64	68.0	56.0
Middle	67	81.6	58.3	113	68.9	62.8
Fourth	113	62.4	52.0	146	73.7	52.2
Highest	16	52.5	34.6+	42	72.2	56.7
**Occupation**						
Unemployed	11	82.1	43.8	83	69.2	53.8
Employed	323	66.0	50.9	399	66.7	45.2
Student	23	56.5	43.9	10	79.4	47.4
**Disability status**						
no difficulty in all domains	261	67.1	50.8	330	69.1	48.6
A lot of difficulty /unable to function in at least one domain	98	63.1	40.8	162	63.9	43.4
**Number of living children**						
0	39	65.0	45.0	12	69.6	40.9
1	39	61.3	51.9	76	59.2	54.0
2	37	60.0	59.4	90	74.8	53.4
3	51	77.7	48.7	75	71.9	45.5
4+	155	65.1	47.7	220	65.2	40.8
**Method currently use**						
IUD	16	81.3	--	12	80.3	--
Implants	42	94.2	--	76	83.6	--
Injectables	35	73.0	--	90	89.9	--
Male condoms	74	76.4	--	75	88.2	--
Pills	14	73.1	--	220	100.0	--
**Total**	359	66.0	48.8	492	67.4	47.1

# Considered exchange rate of $1. 00=3650 Uganda shillings.

* the question is asked to only those that did not pay for their last FP services

+ few observations- only 16 women who attained tertiary education answered on use of own money, only 8 answered on willingness to pay more for FP services and 12 on future use of FP services.

- no observations/not applicable

Nearly half (49%) of men and women (47%) that had never used FP before were willing to pay for FP services in future. Among men the proportion rose from 44% in age group 15–19 to 67% in age group 35–39 but subsequently reduced to 35% in age group 50–54 while among women it rose from 52% in 15–19 age group but rose to 56% in the next age group but reduced to 33% in 45–49 age group. Among men and women, the level was lowest among the uneducated, the disabled and those without children.

### Multivariable analysis of ability to pay for FP services

Table 3 shows results of multivariable linear regression for ability to pay (ATP) measured by natural logarithm of the amount of money the respondents paid for their last FP services. The transformation to natural log is dictated by the normality condition of the linear regression. The factors that independently determined ATP were age group, education level and region of residence. Among men, the amount of money paid increased with age group, was lowest in West Nile and East Central, while among women it reduced with age group, increased with education level, was lower among the disabled, reduced inregions of eastern, east central and Karamoja after controlling for the rest of background characteristics. Specifically, among men. the log payment for those aged 35+ was 0.62 higher compared to those aged 15–24 and in West Nile the log payment was 1.6 lower than that in Central 1. Among women, natural log payment among those who attained tertiary education was 1.6 higher than that of those who did not attain any formal education while in west Nile it was 0.5 less than in Central 1. In summary, the amount of money respondents were able to pay varied significantly by age group, education attainment, region and disability status among women while it varied only by age and region among men.

**Table 3:** Multivariable linear regression analysis for natural log of the amount of money paid for the last FP services

Factors	Men			Women		
	n	Un-adjusted B- coefficient(se)	Adjusted B- coefficient(se)	n	Un-adjusted B- coefficient(se)	Adjusted B- coefficient(se)
**Age group**						
15–24	75	1.0	1	100	1.0	1.00
25–34	115	0.30 (0.17)	0.27(0.18)	128	0.00 (0.11)	-0.11(0.11)
35+	94	0.64 (0.18)***	0.62 (0.18)**	80	-0.25 (0.12)*	-0.25(0.12)*
**Residence**						
Urban	97	1	1.0	87	1.0	--
Rural	187	0.23(0.15)	-0.11 (0.17)	221	-0.36(0.11)**	--
**Education**						
None	8	1	--	34	1.0	1.0
Primary	136	-0.38(0.44)	--	157	0.18 (0.15)	0.18(0.16)
Secondary	118	-0.26 (0.45)	--	109	0.33(0.16) *	0.22 (0.16)
Tertiary	22	-0.03 (0.50)	--	8	1.81 (0.32) ***	1.55(0.32)***
**Region**						
Central 1	68	1.0	1.0	85	1.0	1.0
Central 2	52	0.20(0.21)	0.26 (0.22)	69	-0.35(0.13)*	-0.20(0.13)
Eastern	46	−0.38(0.22)	-0.31(0.23)	44	-0.63(0.16) ***	-0.51(0.15) ***
East Central	43	-0.53(0.22)*	-0.45 (0.22) *	41	-0.43(0.16) **	-0.33(0.15)*
Karamoja	6	0.26(0.48)	0.49(0.49)	4	-1.05(0.42)*	-1.04(0.41)*
West Nile	44	-1.62(0.26) ***	-1.59(0.27) ***	54	-0.21(0.28)	-0.11(0.27)
Western	25	-0.31(0.22)	-0.30 (0.22)	11	-0.12(0.14)	-0.04(0.14)
**Disability**						
No difficulty	214	1.0	1.0	198	1.0	1.0
At least one	70	-0.07(0.17)	-0.18 (0.16)	110	-0.32(0.10)**	-0.20(0.10)*
**Occupation**						
Unemployed	13	1.0	1.0	64	1.0	--
Student	13	-0.28(0.35)	-0.47 (0.33)	9	0.02(0.12)	--
Employed	257	-0.72(0.48)	-0.26(0.45)	235	0.27(0.31)	--
**Wealth** **Index**						
Lowest	35	1.0	--	48	1.0	--
Lower	46	-0.27(0.27)	--	34	-0.08 (0.18)	--
Middle	60	0.37(0.26)	--	74	0.15 (0.15)	--
Higher	128	0.30(0.23)	--	112	0.32(0.14)*	--
Highest	15	0.10(0.37)	--	40	0.66(0.18)***	--
**Number of** **children**						
None	31	1.0	--	10	1.0	--
One	36	0.41(0.30)	--	52	0.27 (0.29)	--
Two	37	0.73(0.30)	--	47	0.05(0.29)	--
Three	41	0.61(0.29)	--	56	0.10(0.28)	--
Four or more	98	0.62(0.25)*	--	123	-0.14(0.27)	--

* p<0.05

** p<0.01

*** p<0.001

NB: This table includes only those who paid for the last FP services they sought. Variables left out of the adjusted model did not make a substantial contribution to the log likelihood of the model. The data in the multivariable models are not weighted.

### Multivariable analysis for willingness to pay for FP in future

Table 4 shows results of MPR analysis for willingness to pay (WTP) for FP in future among men and women. The factors independently associated with WTP among men were being resident in urban areas and from regions of central 2, Eastern and Western regions while among women its being younger (15–24), attainment of primary/secondary education level, being in higher wealth index and being from Eastern region.

**Table 4:** Factors associated with willingness to pay for FP services in future among men aged 15–54 and women 15–49 years in RISE project area

Factors	Men			Women		
	n	Crude PR (95% CI)	Adjusted PR (95% CI)	n	Crude PR (95% CI)	Adjusted PR (95% CI)
**Age group**						
15–24	230	1.0	1.0	320	1.0	1.00
25–34	243	0.87 (0.75–1.00) *	0.98 (0.81–1.18)	284	0.88 (0.77–1.01)	0.94 (0.80–1.11)
35+	242	0.74 (0.63–0.86) ***	0.84(0.68–1.05)	217	0.71(0.60–0.83) ***	0.78 (0.64–0.96) *
**Residence**						
Urban	130	1.0	1.0	148	1.0	1.0
Rural	585	0.74 (0.65–0.83) ***	0.62 (0.51–0.76) ***	673	1.07 (0.91–1.26)	1.17 (0.96–1.42
**Education**						
None	121	1.0	1.0	199	1.0	1.0
Primary	394	1.70(1.32–2.19) ***	1.04 (0.74–1.45)	445	2.27 (1.80–2.86) ***	1.44 (1.13–1.85) **
Secondary	178	2.00 (1.54–2.58) ***	1.12 (0.78–1.61)	166	2.63 (2.07–3.35) ***	1.46 (1.10–1.92) **
Tertiary	22	2.05 (1.44–2.91) **	0.88 (0.50–1.54)	11	2.26 (1.37–3.73) ***	1.00 (0.43–2.29)
**Region**						
Central 1	107	1.0	1.0	111	1.0	1.0
Central 2	99	0.93 (0.79–1.09)	1.68 (1.26–2.22) **	119	1.12 (0.96–1.30)	1.15 (0.95–1.40)
East Central	115	0.85 (0.70–1.03)	1.28 (0.97–1.68)	116	0.91 (0.75–1.10)	1.09 (0.88–1.34)
Eastern	87	0.79 (0.66–0.96)	1.39 (1.03–1.88) *	106	1.07 (0.91–1.26)	1.23 (1.00–1.52) *
Karamoja	119	0.39 (0.29–0.53) ***	0.67 (0.41–1.09)	146	0.26 (0.18–0.37) ***	0.41 (0.25–0.68) ***
West Nile	119	0.60 (0.45–0.80) ***	1.16 (0.79–1.69)	136	0.56 (0.42–0.75) ***	0.81 (0.57–1.14)
Western	69	0.97 (0.82–1.14)	1.57 (1.19–2.06) **	87	0.89 (0.74–1.08)	1.10 (0.87–1.38)
**Disability**						
No difficulty	558	1.0	1.0	556	1.0	1.0
At least one	157	0.93 (0.80–1.09	0.86 (0.70–1.05)	265	1.00(0.88–1.14)	0.98 (0.86–1.11)
**Occupation**						
Unemployed	24	1.0	1.0	167	1.0	1.0
Student	78	1.23 (0.85–1.77)	1.18 (0.75–1.85)	47	0.99 (0.85–1.15)	0.94 (0.80–1.09)
Employed	609	0.99 (0.70–1.40)	1.19 (0.69–2.05)	606	1.44 (1.18–1.74) **	0.89 (0.58–1.36)
**Wealth Index**						
Lowest	286	1.0	1.0	327	1.0	1.0
Lower	98	1.53 (1.26–1.86) ***	1.16 (0.90–1.49)	94	2.28 (1.87–2.77) ***	1.51 (1.22–1.88) ***
Middle	114	1.69 (1.41–2.01) ***	1.34 (1.06–1.69) *	166	2.26 (1.89–2.71) ***	1.46 (1.19–1.79) ***
Higher	196	1.66 (1.41–1.95) ***	1.16 (0.92–1.46)	183	2.19 (1.83–2.62) ***	1.41 (1.14–1.73) **
Highest	21	1.79 (1.36–2.35) ***	1.10 (0.70–1.73)	51	2.34 (1.88–2.91***	1.68 (1.29–2.19) ***
**Number of** **children**						
None	71	1.0	1.0	47	1.00	
One	91	0.98 (0.78–1.23)	1.03 (0.81–1.32)	95	1.17 (0.88–1.56)	1.13 (0.83–1.52)
Two	71	0.89 (0.69–1.15)	1.03 (0.79–1.35)	114	1.02 (0.77–1.37)	1.16 (0.85–1.59)
Three	83	0.86 (0.67–1.10)	1.05 (0.80–1.36)	133	0.96 (0.71–1.28)	1.09 (0.80–1.49)
Four or more	261	0.86 (0.72–1.04)	0.99 (0.76–1.28)	326	0.87 (0.67–1.14)	1.10 (0.79–1.54)

* p<0.05

** p<0.01

*** p<0.001

NB: This table does not include those who did not know or were not sure whether they were willing to pay in the future. The data in the multivariable models are not weighted.

Among men, the prevalence of WTP in future in rural areas was 62% (95% CI: 0.51–0.76) of that in urban areas while among women the prevalence in Karamoja was 41% (95%CI: 0.25–0.68) of the prevalence in central 1. The prevalence for WTP among women was over 40% higher among those who attained primary and secondary education compared to those who never attained formal education. The prevalence of WTP among Women in 2^nd^ or higher wealth quintiles was also over 40% higher compared to those in lowest wealth quintile. In summary, WTP varied significantly by rural/urban residence and region among men while it varied by age group, region, wealth quintile, and education attainment among women.

The significance of some variables was reduced in the multivariable model due to raised multicollinearity among the variables (variance inflation factor-VIF=3.9). The VIF didn't warrant action on the variables included since it was below 1029, 30.

## Discussion

The results on higher affordability and willingness to pay for FP services among women in urban areas, those with higher education and those in higher socio-economic status are similar to findings in a study in Nigeria^31^. Findings on higher willingness to pay (WTP) for FP among middle aged people, those without a disability are consistent with those found in several studies in Asia and Africa 11, 32, 33.

Higher proportion of use of own money to pay for FP services among men compared to women may be explained by generally higher income and employment levels among men than women^34^. A study in Nigeria found out also that men were more likely to have an out-of-pocket expenditure for essential health services than women^35^. Another study found out that in India men were willing to pay for higher prices for FP services than women^36^. Lack of variation by sex for amount paid for contraceptive services, willingness to pay if the cost of FP services was increased by 10% and the general willingness to pay for FP services needs further investigation. Minimal difference between men and women with other indicators of ATP and WTP needs further investigation.

Lower ATP for FP services among those without children may be explained by the association with being young and having low or no income. Most of them (80%) were <=23 years and were single (74%). Forty four percent of these have no occupation or are students.

The large difference between ATP and WTP (85% vs 67%) among men may be attributed to lower sensitization on economic and social benefits of uptake of FP. It has been noted that there is limited accurate knowledge about contraceptive methods among men in Uganda and that fear of side effects is quite prevalent^37^.

The pattern of ability and willingness to pay for FP services across different regions follows the poverty pattern. According to the poverty map for Uganda, Karamoja, Eastern and West Nile are among the poorest regions of the country^38^ and this study shows the same regions have lowest levels of ability and willingness to pay.

Higher proportion that paid at least Ug she 1000 (US cents 27) for last FP services among women compared to men may be explained by what women and men pay for. An example, cheapest methods include male condoms and oral pills and prevalence of current condom use was 36% among men while that for oral pills was 4.5% among women^39^. With such an example the methods women use cost more than the Ug Shs 1000.

The revelation that the respondents' wealth quintile was not consistently associated with the ATP and WTP for family planning shows socioeconomic status alone may not ensure equity in access to FP services. Other studies have confirmed strong association wealth status and ATP and WTP^31^, ^40^.

The high level of willingness to pay for the same FP services in future (79%) is nearly similar to findings in a study in Ghana where more than 75 percent of FP clients were willing to pay at least 50 percent more than they were paying 41. It may be a reflection of high quality of services.

Findings on increasing access to FP services through lowering the cost were nearly similar to studies in Nigeria where with reduction of the cost to US$1.70, more than 75% of consumers were willing to pay for the Progesterone Vaginal Ring^42^. This compares well with results of this study that show that lowering the cost to Ug shs 1000(US cents 27) will ensure access to FP service for 96% of women and 82% of men. For the future, if the cost of FP services is fixed at Shs 2000 (US Cents 54) 93% of women and 83% of men who have never used FP before will access the services.

Differing levels of ATP and WTP for FP services by different groups are evidence for support of the planned TMA to family planning services in Uganda. With the poverty level close to 30% 43 and clear evidence of categories of people that cannot afford to pay in this study TMA may be the best option for more family planning uptake in the country. Appropriate pricing of subsidized and full-cost of the services for those who are able to pay can help to creata robust and healthy market that maximizes demand^44^. An example, services may remain free in the public sector then a nominal cost can be imposed in the commercial sector while the private clinical sector pays higher costs.

## Submission declaration and verification

The work in this paper has not been previously published nor is it being considered for publication anywhere. The publication is approved by all authors and explicitly approved by responsible authorities in the country. If accepted, it will not be published elsewhere in the same form, in English or any other language, including electronically without the written consent of the copyright holder.
